# Strategies for gene disruption in *Drosophila*

**DOI:** 10.1186/2045-3701-4-63

**Published:** 2014-10-14

**Authors:** Shih-Ching Lin, Yu-Yun Chang, Chih-Chiang Chan

**Affiliations:** Graduate Institute of Physiology, National Taiwan University, No.1, Sec. 1, Jen-Ai Rd., Zhongzheng Dist, Taipei, 100 Taiwan; Graduate Institute of Molecular Medicine, National Taiwan University, No.1, Sec. 1, Jen-Ai Rd., Zhongzheng Dist, Taipei, 100 Taiwan; Graduate Institute of Brain and Mind Sciences, National Taiwan University, No.1, Sec. 1, Jen-Ai Rd., Zhongzheng Dist, Taipei, 100 Taiwan

**Keywords:** *Drosophila*, CRISPR, Cas9, Transposons, Homologous recombination, Gene knock-out, Genome editing

## Abstract

*Drosophila melanogaster* has been a classic model organism for the studies of genetics. More than 15,000 *Drosophila* genes have been annotated since the entire genome was sequenced; however, many of them still lack functional characterization. Various gene-manipulating approaches in *Drosophila* have been developed for the function analysis of genes. Here, we summarize some representative strategies utilized for *Drosophila* gene targeting, from the unbiased ethyl methanesulfonate (EMS) mutagenesis and transposable element insertion, to insertional/replacement homologous recombination and site-specific nucleases such as the zinc-finger nuclease (ZFN), the transcription activator-like effector nuclease (TALEN) and the CRISPR (clustered regularly interspaced short palindromic repeats)/Cas9 system. Specifically, we evaluate the pros and cons of each technique in a historical perspective. This review discuss important factors that should be taken into consideration for the selection of a strategy that best fits the specific needs of a gene knockout project.

## Background

*Drosophila melanogaster* is a well-studied model organism. Because 75% of human disease genes have counterparts in the *Drosophila* genome [1, 2], fruit flies has been used as a genetic model to study physiological mechanisms and pathological conditions such as aging [3], diabetes [4], neurodegenerative disorders [5], and cancer [6]. With more than 15,000 annotated genes [7], merely 37% of *Drosophila* genes that have obvious phenotypes are further studied, while the remaining still awaits future investigation [7, 8]. Conventional unbiased forward genetic screens with the employment of ethyl methanesulfonate (EMS) [9] or X-rays [10] have successfully uncovered numerous mutants based on various visible phenotypes. Random-insertion of *P-elements* is another common method to generate mutants by creating deletions following the excision of *P-elements*. As for targeted mutagenesis, fly biologists have mainly relied on two forms of homologous recombination-based gene targeting: insertional (“Ends-In”) [11] and replacement (“Ends-Out) [12], for the past decade. Although the aforementioned approaches are powerful in identifying novel functions of the annotated genes, large-scale genetic screenings via these methods may be both labor-intensive and time-consuming.

In recent years, several sequence-guided DNA endonucleases have been applied to generating targeted mutations in model organisms including *Drosophila* [13]. These site-specific nucleases are programmable, that is, they can induce DNA double-strand breaks (DSBs) that stimulate non-homologous ends-joining (NHEJ) and/or subsequent homologous recombination (HR) at targeted loci [14], therefore generate a frame-shift mutation or a replacement with an extragenous null allele. This type of gene knockout techniques includes the zinc-finger nuclease (ZFN) [13], the transcription activator-like effector nuclease (TALEN) [15], and the CRISPR (clustered regularly interspaced short palindromic repeats)/Cas9 systems [16–18]. Particularly, CRISPR/Cas9 has drawn the attentions of *Drosophila* biologists because it greatly reduces the time and financial requirement, and makes genome-wide gene knockout projects much more practical. Below we will summarize these important approaches in gene disruption, discuss the pros and cons of each technique, and end with emphasis on the applications and future challenges of CRISPR/Cas9 system.

### EMS mutagenesis

The use of potential DNA alkylating agents, especially EMS, has been a standard approach for mutagenesis during the classic era of forward genetic screenings in model organisms. EMS induces new mutations, mostly GC to AT transitions [19], once every ~400 kb at random sites of *Drosophila* genome [20]. As EMS shows no preference for coding sequence, it is able to disrupt genes unbiasedly and to create mutations of various nature in one gene. Besides, the relative low-cost makes EMS the most commonly used reagent to survey all genes in a mutagenesis scheme aiming to saturate the whole *Drosophila* genome [9]. Although the ease of use is appealing, several potential pitfalls of EMS mutagenesis should be taken into consideration. First, its phenotype-based approach narrows the investigations to certain discernible phenotypes, such as the changes of eye colors, organ sizes or wing shapes. In this way, mutations generated by EMS may be either overlooked because of the subtle phenotypes or unable to recover due to the lethality. Second, most cases of EMS-based mutagenesis result in point mutations. For a given gene, a single point mutation may only generate a hypomorphic allele with subtle phenotypes. Therefore, the effects of a gene may be underestimated by simply analyzing the hypomorphic mutants, which may lead to an underestimation in the effects of a gene. Third, mapping the mutations induced by EMS is fairly challenging since the mutated loci are not tagged with recognizable sequences, unlike some other approaches such as *P-element*-mediated disruptions (the details will be discussed later). Besides, EMS often mutates only one strand of the DNA and leaves the unmutated complementary strand, thus causing genetic mosaic in the progeny after rounds of DNA replications [9]. In order to limit the coverage of the genome and to ensure that the phenotype of interest can be transmitted to future generations, screenings following EMS mutagenesis commonly focus on individual chromosomes or chromosome arms. Since the location of an EMS mutation now can be better determined by using high-resolution, high-throughput SNP mapping, identifying the hits from the EMS screening has become relatively promising and more appealing to researchers [21]. Lastly, as EMS may produce multiple hits in the gene of interest [20], verifying the mutation responsible for the scored phenotype requires laborious complementation tests or full-genome sequencing. The time and effort has been considerably reduced nowadays in order to plot the molecular nature of an EMS mutation since the *Drosophila* genome sequence was released and next-generation sequencing became available [20]. Undoubtedly, EMS mutagenesis is still considered a powerful tool for *Drosophila* gene manipulating.

### Transposon-mediated mutagenesis

**Figure 1:**
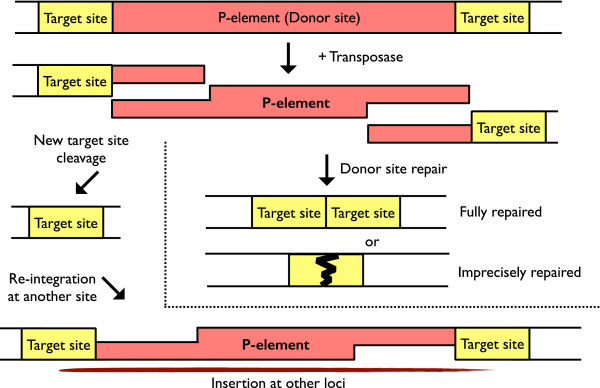
**The scheme of** ***P-element*** **transposition.** The illustrations show a model of *P-element* mediated transposition. First, the transposase binds to sequences within both *P-element* termini and initiates a DSB at each end. The excised *P-element* could be translocated into a new target site to disrupt another gene (left). Gap repair can then generate duplicated target sites with the *P-element* sequences at each end. The 3′ extensions left at the donor site can be used for repair either from homologous sequences located in the other copy as a fully repaired gene which contains two adjacent *P-element* target site (top right), or by non-homologous end-joining for imprecise repair, which could generate products that contain varying lengths of *P-element*-derived sequences as the imprecisely repaired condition (bottom right).

Transposons are pieces of DNA that are mobile in the genomes. Taking the nature of mobility, several transposons have been employed for gene disruption and modification in *Drosophila*, including *P-elements*, *piggyBacs* and *Minos*. Traditionally, *P-elements* are extensively used for forward mutagenesis either by direct gene disruptions following the insertions or via the imprecise excisions after the second translocation events. As the class II transposon, a *P-element* moves in the genome through a DNA-based "cut and paste" mechanism and tends to insert near actively transcribed genes, where the chromatin structure is relatively loose [22]. Each *P-element* encodes a protein called P transposase, which recognizes the inverted repeats of *P-element* and mobilize itself from the original site. An engineered exogenous *P-element* for mutagenesis contains an internal deletion that abolishes the translation of the transposase, but such *P-elements* inserted in the genome can also be mobilized by addition of the transposase gene in the other chromosome via a cross [23]. The mobilized *P-elements* sometimes remove the flanking DNA, creating a deletion where *P-elements* have originally been integrated [24]. Thus, despite *P-element* insertions may not completely disrupt the function of genes; loss-of-function alleles are often generated by the imprecise excision (Figure 1).

The transposon-based gene disruption has several advantages that outrank EMS mutagenesis. First, the occurrence of second-site mutations in each individual is relatively low in the *P-element* system. Visible markers, such as *white*^*+*^, may be engineered to be a part of the transposon, and multi-transposition events can be easily selected out by the presence or absence of markers at the early stages of screening. Second, *P-elements* show high frequency of mobilization, and they can be modulated via the expression of active transposases that are controlled temporally and spatially. Third, the screen efficiency for the transposon-based mutagenesis largely depends on the marker(s) that can be scored. For example, *P-element* insertions with *white*^*+*^, as mentioned above, allow molecular mapping by scoring the eye color. Additionally, the insertion sites could be mapped by calculating the recombination rate between different *P-element* insertions [25], or directly identified by techniques such as inverse PCR [26] or splinkerette PCR [27].

The main disadvantage of *P-element*-mediated gene disruption is its preference for inserting into “hotspots”, which largely reside in the promotor regions of some genes [28]. Regardless of original insertion loci and the nature of sequence composition, 30–40% of the *P-elements* land in the same 200–400 genomic hotspots, making the genome-wide, saturated disruption scheme by *P-element*s less feasible. Interestingly, the preference for hotspots appears to be unique to *P-elements* and is evidently absent in other transposons, such as *piggyBac* or *Minos*, since *piggyBac* favorably targets TTAA sequences while *Minos* inserts at random [29]. Although *P-elements* have once been considered a powerful tool for genome-wide gene disruption, single *P-element* insertions are only able to disrupt 25% of *Drosophila* essential genes and about 40% of the total annotated genes, according to the estimation from the Berkeley *Drosophila* Genome Project (BDGP) [30, 31]. Using *piggyBac* and *Minos* has significantly improved the mutagenesis rate to 60% of the genome [32]. However, the low frequency in *piggyBac*-mediated imprecise excision has hindered them from further creating mutations in the genes of interest directly. Subsequently, the development of *Minos-Mediated Integration Cassette (MiMIC)* for gene-targeting has improved this weakness. In addition to the backbone of *Minos*, *MiMIC* is modified to carry a gene trap cassette flanked by two inverted *ΦC31 attP* sites. With *Minos* showing no insertion site preference, targeting every *Drosophila* gene is feasible and therefore this transposon could potentially accomplish unbiased genome-wide mutagenesis [33]. Taking advantage of *ΦC31 attP* sites, gene trap cassettes in *MiMIC* can be easily swapped with any DNA sequence flanked with *attB* sites by *ΦC31* recombinase, allowing further gene modifications and genome editing [33]. For examples, a florescence reporter can be introduced to tag the protein expression and the effects of *MiMIC* insertions can be reverted after excising the whole cassettes.

### Homologous recombination

Mutagenesis via chemicals or transposons is used to create mutations at random sites of the genome, and it has to be followed with screenings to identify phenotypes of interests. This type of classical forward genetic approach has proved its way in identifying novel genes while, in the opposite way, a gene can be specifically mutagenized in a reverse genetic approach once the genome sequence is available. The classical reverse genetic approach for *Drosophila* utilizes the homologous recombination (HR) to replace genomic DNA. HR regularly occurs at low frequencies in normal cells and it repairs DSBs after DNA damages thus increases genetic variation during meiosis [34]. Some microbes also employ homologous recombination to exchange genetic material between different strains [35].

**Figure 2:**
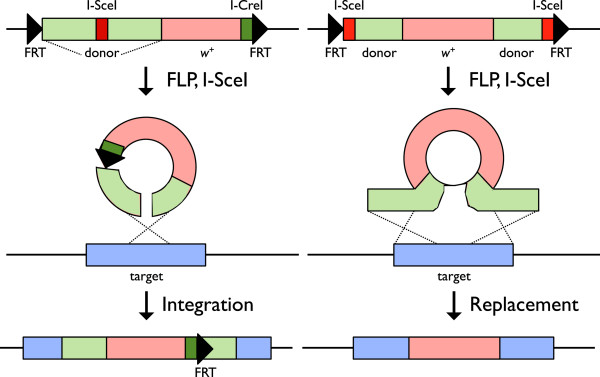
**The comparison of Ends-In and Ends-Out homologous recombination.** Ends-In and Ends-Out are two paradigms for gene targeting. The major difference is whether the DSB is located within the region of homology (Ends-In) or at the ends (Ends-Out). The figure compares the basic outcomes of these two methods. With ends-in (left), a break is made within the region of homology. Recombination with the target results in a tandem duplication of all the homologous sequence carried on the donor, separated by any sequences that are between the *FRT* sites (in this case, the *white* ^*+*^ and *I-CreI* site). In contrast, Ends-Out provides a simple replacement event between the genome and the homologous sequence. The result is to interrupt the targeted gene with a modified, heterologous sequence, such as the *w* ^*+*^ marker (right).

The HR-mediated gene disruption first requires a fly line harboring a donor DNA that is derived from a *P-element* cassette, carrying DNA sequences homologous to the target locus flanked *FLP* Recombinase Target sites (FRTs) and *I-SceI* recognition site(s). Two enzymes, the site-specific recombinase *FLP* and endonuclease *I-SceI* [36, 37], are subsequently introduced into the *Drosophila* line in order to create DSBs in an inserted donor transgene. The recombination between two FRT sites excises the cassette out from the original site and the cleavage at the *I-SceI* recognition site(s) creates a DSB, which induces the homology repair machinery that results in HR between the donor DNA and the targeted chromosomal sequence [38]. The arrangement of homologous sequences and *I-SceI* sites in the donor DNA defines two targeting strategies, Ends-In and Ends-Out, creating a tandem duplication or deletion of the target gene, respectively (Figure 2). Although the Ends-Out targeting is successfully used in mice for gene deletion, the frequency of Ends-Out targeting is relatively low in *Drosophila* [39, 40]. Ideally, candidates are selected out by losing FRT sites; however, high false positives are also documented due to damaged FRTs that are not ruled out by the scoring method and interfere with the efficiency of gene targeting [39]. A modified donor DNA expressing *reaper*, a cell death gene, can eliminate progenies that did not undergo recombination events and therefore speed up the screening process [39]. Addition of positive selection markers, such as the eye-specific 3xP3-RFP to facilitate the screening, can also increase the recovery of correct knockout events [41]. On the other hand, genome editing can be fulfilled by introducing the donor DNA, carrying a desired modification to the site of recombination via the Ends-In targeting strategy [42]. Combined the integration of *attP* site into the gene of interest, repeated genome editing of that gene can be accomplished through the following runs of *ΦC31*-dependent recombination events [43]. Although it is labor-intensive, this Ends-In homologous recombination has been a reliable method for *Drosophila* genome editing.

Because of the limitations in the replication and transposition of *P-elements*, the size of donor DNA is limited to approximately 30 kb. The *P[acman]* vector harbors dual replication origins for maintaining large DNA sequences and for inducing to high copy numbers. Therefore, *P[acman]* can be used to construct donor DNA up to 100 kb via recombineering-mediated gap repair using *Bac* or *P1* genomic clones as templates [43]. Additionally, *ΦC31* integrase-mediated transgenesis significantly increases the efficiency of integrating *P[acman]* plasmid into the fly genome. Further modifications have advanced the applications of *P[acman]* in gene targeting and alterations. For examples, the employment of the Gateway system not only facilitates the tagging, but also provides a platform to knock out genes systematically; the introduction of selection markers, such as the eye-specific 3xP3-RFP that mentioned above, eases the screening process; and the combination of *Cre-loxP* systems allows the removal of the targeting cassettes and therefore reverts the mutations [44].

Although *I-SceI*-mediated homologous recombination opens the door for scientists to disrupt target genes specifically, the low recombination frequency and numerous false-positives have limited this technique from large screenings. Newer methods, including ZFN, TALEN and CRISPR/Cas9, involve the creating DSBs by nucleases directly in genomic DNA, different from the DSBs generated by *SceI* that are created in extrachromosal donor DNA. In the presence of genomic DSBs, different repair machineries are employed based on the availability of the homologous template. NHEJ functions by directly joining the two ends of a DSB is the favored way when a homologous template is absent [45]. Because NHEJ-dependent repair ignores DNA deletion or insertion in DSB sites, frame-shift mutations are sometimes created [46]. Alternatively, DSBs can also be repaired through HR either relying on the other copy of undamaged chromosome in a diploid organism or using an exogenous DNA fragment as a template. In the latter case, a donor DNA sequence with homology arms is specifically introduced into the target gene, which results in gene modification in a base pair-precise manner [47].

### ZFN and TALEN

**Figure 3:**
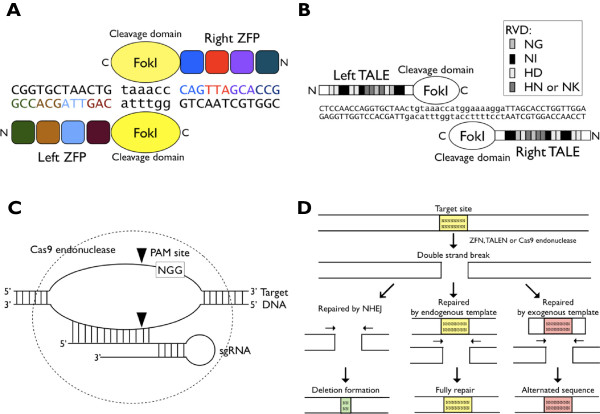
**Site-specific endonucleases.** Three classic site-specific endonucleases including ZFN, TALEN and CRISPR/Cas9 are shown. **(A)** ZFN simply consists of a Zinc-finger protein (ZFP) fused to *Fok1* endonuclease. The sequence composition of the α-helix in the zinc-finger determines the nucleotide binding specificity of the ZFP. As a result, a ZFP chain can be created by joining a few ZFPs together and generating high specificity, allowing *Fok1* endonuclease to accurately cleave DNA at the target site. **(B)** The C-terminal end of a TALE contains a *Fok1* endonuclease for DNA cleavage. The central part of the TALE contains a number of almost similar repeats that mediate specific binding to target loci in the genome, and each of these repeats specifically binds to one base of the target DNA via two amino acids named repeat variable di-residues (RVDs), including NG, NI, HD and HN (or NK) for recognizing one of the four different nucleotides: T, A, C and G, respectively [53, 54]. **(C)** Cas9 forms a sequence-specific endonuclease when complexed with the sgRNA. The Cas9/sgRNA complex then recognizes the targeted sequence, 20-bp in length, ending with two guanines (NGG) called the PAM site. Cleavage occurs on both strands upstream of the PAM sites. **(D)** The DSB is first induced by ZFN, TALEN or Cas9 endonuclease and then repaired by three possible mechanisms. When repaired by NHEJ, random deletions would occur at the site (left). When the repair is done by the endogenous template within the genome, the sequence would be fully repaired (middle). If an exogenous modified template is added, the sequence could be altered after repair, which is regarded as the gene editing (right).

ZFN and TALEN are artificial chimeric enzymes that contain a DNA binding domain and a *Fok1* DNA-cleavage domain (Figure 3A-B). With the DNA cleavage action itself showing no sequence preference, the specificity of ZFN and TALEN is determined by their DNA-binding domains, which can be engineered to recognize specific sequence in the gene of interest. Each Zinc-finger domain consists of approximately 30 amino acids that bind to 3 base pairs of nucleotides. Instead, TALEN uses the DNA-binding domains from transcription activator-like effectors (TALEs) and recognizes a single base pair. Therefore, linking multiple Zinc-fingers or TALE repeats are required to create a long DNA recognition sequence and increase the specificity of gene targeting. The mutagenesis efficiency of ZFN-induced gene targeting in *Drosophila* has been estimated to be as high as 1–10% at several loci including *ry, coil* and *pask* genes [47, 48]. A test at the *y* locus via ZFN-targeting has proved that DSBs can enhance the efficiency of gene targeting over simply homologous recombination [47]. ZFN system has evidently facilitated researchers in the aspect of manipulating the genes of interest; however, in *Drosophila* and other organisms such as the zebrafish [49] and *Caenorhabditis elegans* [50], the efficiency of TALEN is superior to that of ZFN. Considering the fact that each TALE targets a single nucleotide, it provides better design flexibility than that of a Zinc-finger. Moreover, the length of spacers between DNA-binding sites is less flexible in ZFN than those in TALEN that show less effect on nuclease activity with either an extra or deleted base pair. The specificity of ZFN depends on the affinity between the zinc-finger domain and the target site; therefore, ZFN has to be designed for each specific target. The short binding sites of ZFN increase the possibility of off-target cleavage, which might result in elevated DSBs events in the cells and cause cell death [51]. Even though TALEN outranks ZFN in many ways, it is speculated that TALEN does not release the ends immediately after cleavage and may interfere with the onset of DNA repair [52].

### Cas9, sgRNA mediated high specificity for gene interruption

Although ZFN and TALEN have demonstrated significant advances in the technology of gene targeting, they still require a new design for each gene, unaccommodating for systematic gene-disruption. CRISPR/Cas9 uses a small guide RNA (sgRNA) to target DNA, dramatically lowers the difficulties for site-specific gene modification. CRISPR/Cas9 is currently considered the most popular tool in the genome-editing era. The system, which originated as a component of prokaryotic innate immunity system [55], recognizes target loci via sgRNA sequence: this 20-bp sgRNA requires an adjourning NGG sequence known as the protospacer adjacent motif (PAM) site for Cas9 recognition [56–58] (Figure 3C). In brief, the editing efficiency of Cas9 to a target gene is deeply related to the selection of sgRNA, including the distance between its cleavage site and the translation start site, the GC content, the strand selection and the last four nucleotides before the PAM site of the sgRNA. All of the factors listed here should be taken into consideration to enhance the performance of Cas9-mediated genome editing [59].

Cas9 has grown in popularity for *Drosophila* gene modification because of the following advantages over other methods. First, both ZFN and TALEN require the engineering of proteins for the specificity of gene targeting; instead, a change in 20-bp sgRNA is sufficient for Cas9 to distinguish genes and NGG sequences are abundant in the fly genome. Second, several methods have been tested to deliver the Cas9 system to fly embryos: (1) introducing of plasmid vectors or *in vitro* transcribed RNA encoding Cas9 and sgRNA [60]; (2) injecting sgRNA plasmid DNA into Cas9 transgenic flies, which significantly increases the editing flexibility and efficiency [17]; (3) establishing a transgenic model of Cas9-sgRNA complex, which shows higher efficiency and stability [61]. Third, similar to *P[acman]*, the introduction of selection markers, such as coinjection of a donor vector carrying 3xP3-RFP (or 3xP3-DsRed)*,* with Cas9 can speed up the screening process and Cas9 can create conditional knock-out lines when combined with the *Cre-loxP* system [60, 62]. These exogenous DNA (3xP3-RFP/3xP3-DsRed or loxP sequence, for examples) are flanked with homology arms about 1 kb long in both ends on the donor plasmid for efficient knock-in [60, 62], and in this way, Cas9-based knock-in has shown better successful rate compared with previous Ends-In techniques [42]. Furthermore, Cas9 can be used as an alternative tactic to create deletion mutations from the pre-existing *P-element* insertions [60]. With the efficiency up to 88% of injected embryos having mutations, Cas9 can generate mutations rapidly [16]. One thing worthy of attention is the off-target effects when using Cas9-based approaches. The target binding capacity of Cas9 remains the same with 1-3 bp mismatches in target sequence unless mutations are located in the PAM domain. This is significantly different from that of TALEN, which can only tolerate 1-2 bp mismatches [57]. Even though both TALEN and Cas9 can accommodate some mismatches in target sequence, it has been shown that Cas9 has more off-target problems in the mammalian genome [63, 64]. High concentration of sgRNA has been shown to impair Cas9 targeting [64]. It is possible that, in some cases, the off-target effects may outweigh the ease of construct design of the Cas9 system. Different gene disruption strategies should be carefully compared before each project based on the context of targets and the goal of the project.

## Conclusions

Since Thomas Morgan and follow researchers have characterized and studied the heritable mutants of thousands of fruit flies, *Drosophila* has served as a key model system in a wide range of biological researches. The new resources and strategies for gene disruption will undoubtedly contribute to the understanding at the systematic complexity of the gene networks in this small yet sophisticated organism, and to the molecular mechanisms underlying these important biological processes. The pros and cons are discussed above. EMS mutagenesis provides an unbiased screen platform but is labor-intensive. *P-elements* are easy to trace and to manipulate, but their preference for insertion sites is far from being random. The method involving HR is site-specific but relatively less efficient; however, the combination of ZFN, TALEN or CRISPR/Cas9 with homologous recombination has made the editing efficiency more promising. While gene targeting is simplified by the use of CRISPR/Cas9, it is now feasible to establish a disrupting library that covers all annotated protein-coding genes in *Drosophila melanogaster*. With the goal of producing a whole-genome mutant collection, scientists have declared the capacity to generate deletion mutants in 50–100 genes per month with an average turnaround time of two months [65]. With the combined efforts of multiple laboratories, it would be possible to generate all of the mutants in a few years, and permit the functional dissection of individual genes in clean genetic knockout background.

The manipulation of exogenous DNA templates in the Cas9 cleavage site could be versatile (Figure 3D). This “knock-in” process allows the generation of a conditional knockout allele by introducing *loxP* sites, the analysis of the expression pattern by inserting a traceable marker, or the generation of point mutations in the endogenous locus for the structure-function analysis. Together, these genetic tools are valuable assets for researchers not only to uncover the physiological functions of *Drosophila* genes, but also to offer insights to decipher the complex mechanisms of human biology and diseases in the future.

## Abbreviations

EMS: Ethyl methanesulfonate
ZFN: Zinc-finger nuclease
TALEN: Transcription activator-like effector nuclease
CRISPR: Clustered regularly interspaced short palindromic repeats
DSBs: Double-strand breaks
NHEJ: Non-homologous end-joining
HR: Homologous recombination
BDGP: Berkeley *Drosophila* Genome Project
*MiMIC*: *Minos-Mediated Integration Cassette*
FRTs: *FLP* Recombinase Target sites
sgRNA: Small guide RNA
PAM: Protospacer adjacent motif
ZFP: Zinc-finger protein
RVDs: Repeat variable di-residues.
